# Steel slag amendment impacts on soil microbial communities and activities of rice (*Oryza sativa* L.)

**DOI:** 10.1038/s41598-020-63783-1

**Published:** 2020-04-21

**Authors:** Suvendu Das, Hyo Suk Gwon, Muhammad Israr Khan, Seung Tak Jeong, Pil Joo Kim

**Affiliations:** 1 Institute of Agriculture and Life Sciences, Gyeongsang National University, Jinju, 660-701 Republic of Korea; 2 Division of Applied Life Science, Gyeongsang National University, Jinju, 660-701 Republic of Korea

**Keywords:** Soil microbiology, Element cycles

## Abstract

With the increase in iron/steel production, the higher volume of by-products (slag) generated necessitates its efficient recycling. Because the Linz-Donawitz (LD) slag is rich in silicon (Si) and other fertilizer components, we aim to evaluate the impact of the LD slag amendment on soil quality (by measuring soil physicochemical and biological properties), plant nutrient uptake, and strengthens correlations between nutrient uptake and soil bacterial communities. We used 16 S rRNA illumine sequencing to study soil bacterial community and APIZYM assay to study soil enzymes involved in C, N, and P cycling. The LD slag was applied at 2 Mg ha^−1^ to Japonica and Indica rice cultivated under flooded conditions. The LD slag amendment significantly improved soil pH, plant photosynthesis, soil nutrient availability, and the crop yield, irrespective of cultivars. It significantly increased N, P, and Si uptake of rice straw. The slag amendment enhanced soil microbial biomass, soil enzyme activities and enriched certain bacterial taxa featuring copiotrophic lifestyles and having the potential role for ecosystem services provided to the benefit of the plant. The study evidenced that the short-term LD slag amendment in rice cropping systems is useful to improve soil physicochemical and biological status, and the crop yield.

## Introduction

Over the past decades, steel production has increased and consequently, the higher volume of by-products (slag) generated necessitates its efficient recycling. In order to mitigate environmental consequences caused by the disposal of a large quantity of slag at landfill sites and to reduce the transportation cost for disposal of slag, steel industries encourage sustainable use of slag in different fields of application, such as in agriculture^1,2^. Slags are rich in Silicon (Si) and other fertilizer components, such as Ca, Fe, Mn, and P^2–4^. These beneficial properties of the slag can be exploited to improve agricultural productivity. For instance, it has been postulated that the adequate Si supply improves tissue rigidity and boosts plant photosynthesis capacity and increases biotic and abiotic stress resistance^5^. The liming nature of slag increases soil pH and helps in nutrient mobilization and improves plant productivity^1^.

Rice (*Oryza sativa* L.) is a Si accumulating plant which can accumulate Si above 10% of shoot dry weight^5^. Silicon is an agronomically essential or quasi-essential element for improving the quality and yield of rice^6^. Intensive rice cultivation chronically depletes soil Si content, consequently imposes negative impacts on soil quality and crop productivity. In recent years, a number of studies have reported the beneficial effects of slag and/or slag-based fertilizer application to improve soil quality and plant productivity, mitigate greenhouse gas emissions from rice cropping systems^7–11^, and stabilize heavy metals in contaminated soils^12,13^.

A mechanistic understanding of soil health indicators is unanimously recognized as a key to improve crop productivity and sustainability^14^. The rhizosphere microbiome plays a pivotal role in nutrient cycling and thus crop productivity^15^. Rhizosphere microbial communities shift with changes in root exudate profiles, rhizosphere carbon and nutrient flows and cumulative plant impacts on the soil environment^16^. Moreover, the variation in the plant nutrient uptake has been reported as a key variable shaping rhizosphere microbial community and activities^16^. In recent years, considerable number of studies highlighted the importance of soil microbiome in ecosystem functioning under different agronomic management practices. For instance, earlier studies reported that the organic manure application as opposed to synthetic fertilizer significantly increased beneficial microorganisms that help in improving soil quality and productivity^17–20^. Reports on the response of soil microbial community structure, activities, and the influencing factors in the slag amendment cropping systems are rare. Besides, plant genotype may influence the shift in rhizosphere microbial community composition owing to the differences in plant photo-assimilates^21^. Our understanding on how rhizosphere bacterial communities and activities are affected by the slag amendment and by plant genotypes (i.e., Indica vs. Japonica rice genotype), and how the changes in microbial communities and activities influence soil quality and productivity are, however, not clear. In our previous study^22^, the taxonomic and functional responses of soil microbial communities to a commercially available slag-silicate fertilizer amendment in rice cropping systems were studied. In this study, we evaluate the amendment impact of Linz-Donawitz converter (LD) slag, generated from the basic oxygen furnace (BOF) process in steel manufacturing, on soil bacterial communities, soil enzyme activities, soil physicochemical changes, and plant nutrient uptake in two different geographic races of *Oryza sativa* var. Japonica (Japonica rice) and var. Indica (Indica rice).

The hypotheses that may primarily determine how the slag amendment affects rhizosphere microbial community and ecosystem services are, (1) as a rich source of fertilizer components, the slag amendment increases nutrient availability not only to the plant but also to the rhizosphere microorganisms and enhances their activities^22,23^, (2) as a rich source of Si, slag amendment increases photosynthesis and belowground carbon allocation, which fuels microbial community and activities^22,23^, and (3) as a liming agent, the slag amendment increases the soil pH towards neutrality (pH 7.0) and improves nutrient availability to the plant which in turn increases belowground microbial activity^23^. Nonetheless, slags may contain traces of heavy metals^24^ that may impede rhizosphere microbial diversity and functionality.

The aim of the study was to elaborate the effects of the slag amendment on soil nutrient status, plant nutrient uptake and growth parameters, soil enzyme activities, and rhizosphere bacterial community structure in rice cropping systems. In this study, we explicitly evaluate whether the measured variables would cause a shift in soil bacterial community composition that reflect in sustainable crop production by the slag amendment in a rice cropping system.

## Results

### Physicochemical characteristics of LD slag

The major elements of the Linz-Donawitz converter slag (LD slag) were Ca, Fe, Al, Si and Mn, while the minor elements were As, Ba, Cd, Cr, Pb, Se, Ag, Sb, Ni, Zn, and Co (Supplementary Table S1). The ASTM (American Standards for Testing Materials) water leachate and the TCLP (Toxicity Characteristic Leaching Procedure) leachate of most of the heavy metals in the LD slag were very low or negligible (Table S1). The comparison of TCLP leachate concentrations of the LD slag with that of TCLP criteria recommends that the LD slag did not exceed U.S. EPA standards for considering whether the slag is environmentally hazardous. The low ASTM concentrations of metals in the LD slag further suggest that the leachate from the slag is less likely to affect groundwater above drinking water standards^11,24^. Whereas, the low TCLP value in the LD slag indicates that the extracted concentrations of metals from the slag in acidic conditions of intestine are negligible when ingested incidentally^24^.

### Soil and plant properties and the crop yield

The LD slag amendment markedly increased soil organic carbon (SOC) by 11.6 and 14.6%, readily mineralizable carbon (RMC) by 37.3 and 42.7%, microbial biomass carbon (MBC) by 26.2 and 30.3%, available phosphorous (AP) by 33.2 and 33.0%, water soluble Si (aqSi) by 898 and 718%, water soluble Fe (aqFe) by 160 and 183%, exchangeable Ca^2+^ by 47.3 and 46.9%, exchangeable Mg^2+^ by 60.2 and 65.0%, whereas decreased ninhydrin reactive nitrogen (NRN) by 15.4% and 16.8% in Japonica and Indica cultivar, respectively. In addition, it increased the photosynthetic rate by 21.1 and 18.0%, straw N, P, and Si content by 20.1 and 22.2%, 17.0 and 18.4%, and 29.9 and 30.5%, grain yield by 15.2 and 13.6%, straw biomass by 19.9 and 22.0%, and root biomass by 17.2 and 19.4%, in Japonica and Indica rice cultivar, respectively (Table 1).

**Table 1:** Effects of LD slag amendment on soil biochemical properties, photosynthetic rate, and yield attributes of Japonica and Indica rice cultivars.

Parameters	Japonica		Indica		Statistical analysis		
	Control	+ LD slag	Control	+ LD slag	Slag	Cultivar	Slag × Cultivar
Soil pH	5.95 b	6.06 a	6.05 b	6.20 a	6.91 ^*^	1.18 ^ns^	0.09 ^ns^
Soil Eh	−64.0 a	−67.0 a	−68.0 a	−72.0 a	0.06 ^ns^	1.37 ^ns^	0.72 ^ns^
SOC (g kg^−1^)	27.6 b	30.8 a	28.1 b	32.2 a	8.84 ^*^	0.54 ^ns^	0.13 ^ns^
MBC (mg kg^−1^)	93 b	496 a	412 b	537 a	81.6 ^***^	5.34 ^*^	0.47 ^ns^
RMC (mg kg^−1^)	177 b	243 a	192 b	274 a	106 ^***^	11.6 ^*^	1.78 ^ns^
NRN (mg kg^−1^)	5.52 a	4.67 b	5.66 a	4.71 b	39.8^***^	0.38 ^ns^	0.13 ^ns^
AP (mg kg^−1^)	76.6 b	102 a	74.8 b	99.5 a	46.1 ^***^	0.49 ^ns^	0.03 ^ns^
_aq_Si (µM)	51.8 b	517 a	55.7 b	456 a	725 ^***^	53.1 ^***^	8.08 ^*^
_aq_Fe (µM)	175 b	456 a	121 b	343 a	259 ^***^	48.6 ^***^	7.38 ^*^
K^+^ (cmol^+^ kg^−1^)	1.16 a	1.37 a	1.13 a	1.30 a	2.34 ^ns^	0.14 ^ns^	0.02 ^ns^
Ca^2+^ (cmol^+^ kg^−1^)	6.23 b	9.18 a	6.27 b	9.21 a	123 ^***^	0.04 ^ns^	0.003 ^ns^
Mg^2+^ (cmol^+^ kg^−1^)	1.33 b	2.13 a	1.20 b	1.98 a	30.7 ^***^	0.14 ^ns^	0.02 ^ns^
^†^Photosynthetic rate (µ mol m^-2^ s^−1^)	20.3 b	24.6 a	22.3 b	26.3 a	16.6 ^**^	1.21 ^ns^	0.06 ^ns^
Grain yield (g pot^-1^)	8.16 b	9.40 a	7.63 b	8.67 a	14.3 ^**^	5.45 ^*^	0.12 ^ns^
Straw yield (g pot^−1^)	27.6 b	33.1 a	38.4 b	46.8 a	6.37 ^*^	19.7 ^**^	0.28 ^ns^
Above ground biomass (g pot^−1^)	35.8 b	42.5 a	46.0 b	55.5 a	10.1 ^**^	20.5 ^**^	0.29 ^ns^
Root biomass (g pot^−1^)	7.41 b	8.69 a	11.4 b	13.6 a	5.83 ^*^	0.11 ^ns^	0.008 ^ns^
Straw N content (mg g^−1^)	4.63 b	5.57 a	4.80 b	5.87 a	21.4 ^***^	1.16 ^ns^	0.09 ^ns^
Straw P content (mg g^−1^)	1.29 b	1.51 a	1.32 b	1.56 a	18.3 ^***^	1.19 ^ns^	0.11 ^ns^
Straw K content (mg g^−1^)	12.3 a	13.1 a	13.9 a	14.8 a	2.24 ^ns^	0.21 ^ns^	0.011 ^ns^
Straw Si content (mg g^−1^)	32.9 b	42.7 a	34.8 b	45.5 a	135 ^***^	6.23 ^*^	0.23 ^ns^

Means of three replicates per treatment are presented. Values in the same row under different cultivar followed by different letters are significantly different at P < 0.05 according to Tukey’s HSD test. In ‘Statistical analysis’ F value followed by significant label (*P*) are provised. ***, **, *, and ns indicates Significant at *P* < 0.001, at *P* < 0.01, at *P* < 0.05, and non significance, respectively. RMC, readily mineralizable carbon; NRN, ninhydrin nitrogen content; AP, available P, aq, water soluble. †Average photosynthetic rate estimated at 42, 70, and 105 DAT (Fig. S1).

The nutrient uptake calculated from the dataset, although partial, magnifies the contrasts between treatments: (i) large increase of nutrient uptake with slag, (ii) large difference in nutrient uptake between Indica and Japonica species, even without slag amendment, (iii) the uptake of K is more contrasted among rice species than with slag, Si uptake is more contrasted with slag than with rice species, while the rice species and slag effects are similar for N and P (Table 2).

**Table 2:** The LD slag amendment effects on nutrient uptake of rice straw.

	Japonica		Indica		Indica/Japonica
	Control	+ LD slag	Control	+ LD slag	IN CONTROL
Straw yield (g pot^−1^)	27.6	33.1	38.4	46.8	
N content (mg g^−1^) straw	4.63	5.57	4.8	5.87	
N uptake (mg, straw only)	128	184	184	275	44%
**Ratio N (slag/control)**		**44%**		**49%**	
Si content (mg g^−1^) straw	32.9	42.7	34.8	45.5	
Si uptake (mg, straw only)	908	1414	1339	2132	
Si uptake (g, straw only)	0.91	1.41	1.34	2.13	47%
**Ratio Si (slag/control)**		**44%**		**49%**	
P content (mg g^−1^) straw	1.29	1.51	1.32	1.56	
P uptake (mg, straw only)	36	50	51	73	42%
**Ratio P (slag/control)**		**40%**		**44%**	
K content (mg g^−1^) straw	12.3	13.1	13.9	14.8	
K uptake (mg, straw only)	340	434	532	694	
K uptake (g, straw only)	0.34	0.43	0.53	0.69	56%
**Ratio K (slag/control)**		**28%**		**30%**	

### Soil enzyme activities

**Figure 1:**
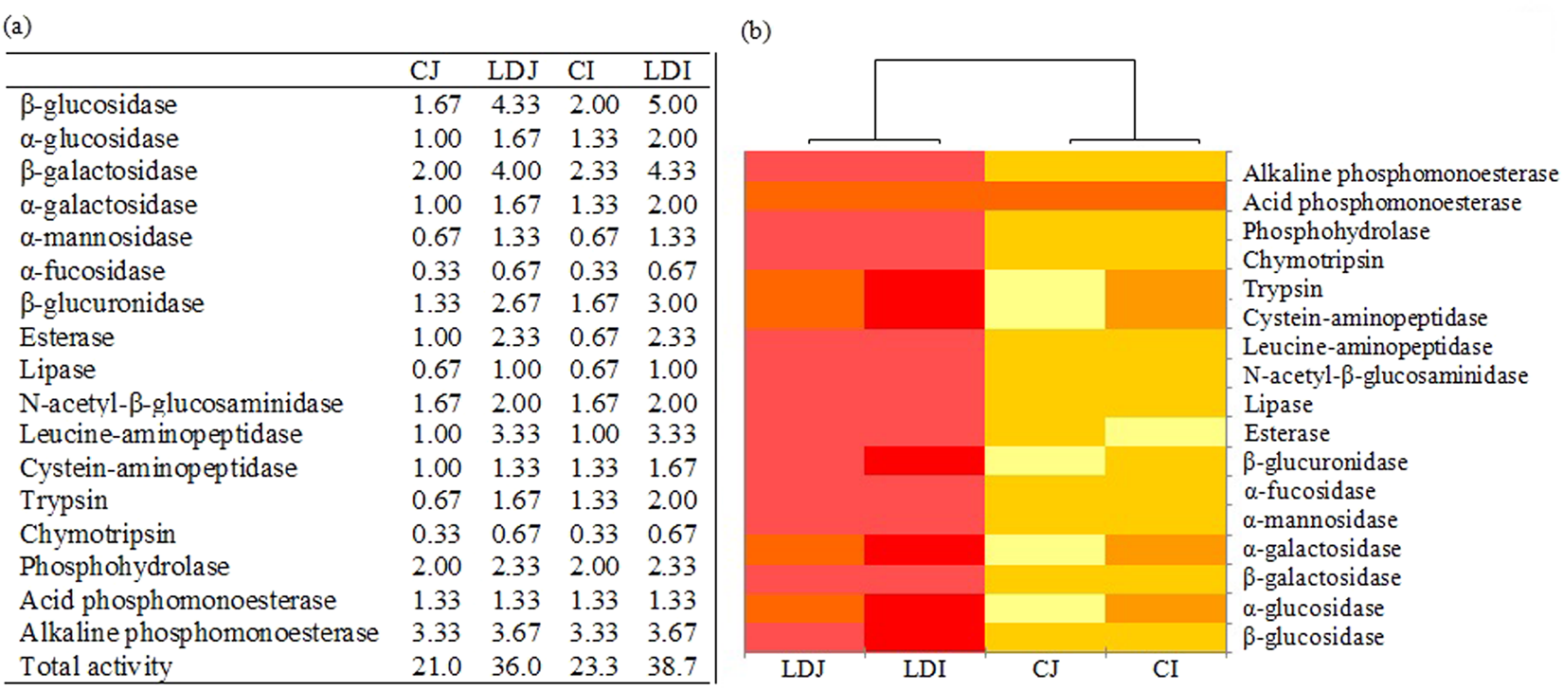
(**a**) Enzymatic profiles of soil based on the hydrolytic activities assessed by the APIZYM system. Values are the mean of triplicate observations, (**b**) Heatmap depicting significant differences in soil enzyme activities among the treatments. The relative abundance of soil enzyme activities is depicted by color intensity. The relative abundance of soil enzyme activities in different samples is colored in shades of yellow (low relative abundance) to red (high relative abundance) through orange. CJ, Japonica without slag; LDJ, Japonica with slag; CI, Indica without slag; LDI, Indica with slag.

Labile carbon degrading enzymes such as α-glucosidase, β-glucosidase, α-galactosidase, β-galactosidase, α-mannosidase, and α-fucosidase which can degrade maltose, cellobiose, melibiose, lactose, mannose, and fructose, respectively, markedly increased in the slag amendment treatments in comparison to the unamended treatments, irrespective of rice cultivars (Fig. 1). Likewise, recalcitrant carbon degrading enzymes such as esterase, lipase, and N-acetyl-β-glucosaminidase which can degrade hemicelluloses and polysaccharide respectively, increased by the slag amendment. Notably, the increase was more in labile carbon degrading enzymes (mostly β-glucosidase, and β-galactosidase) compared to the recalcitrant carbon degrading enzymes. The LD slag amendment also noticeably increased soil enzyme activities involved in nitrogen cycling (i.e., leucine-aminopeptidase and trypsin) and phosphorus cycling (i.e., alkaline phosphomonoesterase and phosphohydrolase). Notably, the increase in soil enzyme activities was more prominent in Indica in comparison to Japonica rice variety. Among the C cycling enzyme, β-glucosidase, and β-galactosidase were dominant, whereas among the N cycling enzyme, aminopeptidase and among the P cycling enzyme, alkaline phosphomonoesterase were dominant in the paddy soil (Fig. 1).

### Relative abundance and diversity of rhizosphere bacterial community

**Figure 2:**
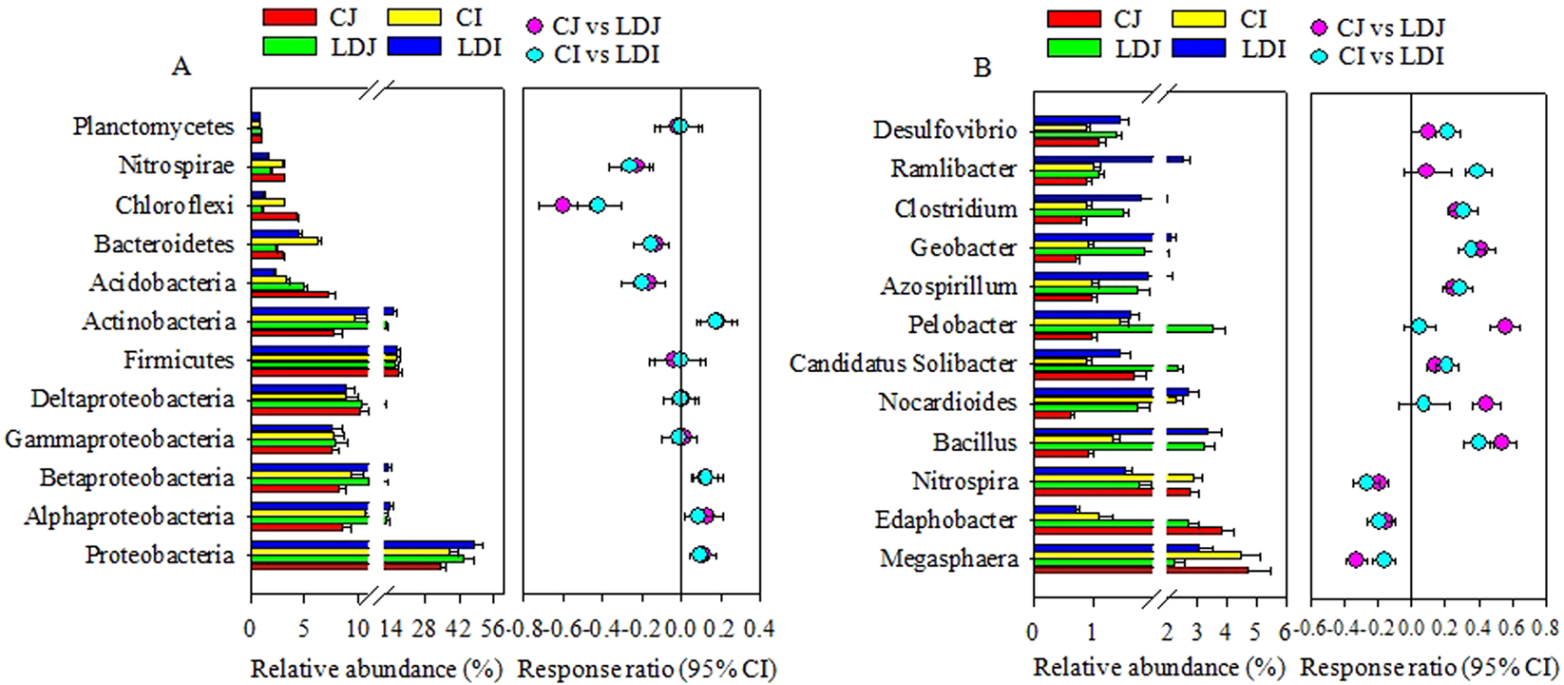
The relative abundance of major (≥1.0%) phylogenetic groups (**a**) and genera (**b**) in the rice rhizosphere as influenced by the LD slag amendment. The relative abundance is presented in terms of percentage in total bacterial sequences per sample. Significantly altered phylogenetic groups and genera were presented in term of response ratio at 95% confidence interval (CI). CJ, Japonica without slag; LDJ, Japonica with slag; CI, Indica without slag; LDI, Indica with slag.

The most abundant (≥ 1.0%) bacterial phyla across treatments were Proteobacteria (34-48%), Fermicutes (15–17%), Actinobacteria (7–18%), Acidobacteria (3–7%), Bacteroidetes (2-6%), Nitrospirae (2–3%), and Chloroflexi (1–4%). The LD slag amendment significantly (*P* < 0.05) increased the relative abundance of Proteobacteria by 28.9 and 25.2%, and Actinobacteria by 52.2 and 50.7%, while significantly (*P* < 0.05) decreased the relative abundance of Acidobacteria by 31.4 and 36.1%, Bacteroidetes by 25.3 and 29.2%, Nitrospirae by 40.6 and 45.4%, and Chloroflexi by 74.7 and 61.9% in Japonica and Indica rice, respectively (Fig. 2). Within Proteobacteria, the relative abundance of Alphaproteobacteria and Betaproteobacteria significantly (*P* < 0.05) increased, whereas the LD slag amendment did not change Deltaproteobacteria and Gammaproteobacteria significantly, irrespective of rice cultivars (Fig. 2). The cultivar, and interaction of LD slag and cultivar did not significantly affect the relative abundance of dominant bacterial phyla (Table S2).

A total of 613 bacterial genera were detected in the rhizosphere of rice. The relative abundance of genera varied greatly across the treatments. The major dominant (>1.0%) genera were presented in Fig. 2. The LD slag amendment significantly (*P* < 0.05) increased the relative abundance of *Bacillus*, *Candidatus Solibacter*, *Azospirillum*, *Geobacter*, and *Clostridium*, while significantly (*P* < 0.05) decreased the relative abundance of *Megasphaera*, *Edaphobacter*, and *Nitrospira* in both the rice cultivars (Fig. 2). However, among the dominant genera *Nocardioides*, and *Pelobacter* significantly (*P* < 0.05) increased only in Japonica rice cultivar, whereas *Ramlibacter*, and *Desulfovibrio* significantly (*P* < 0.05) increased only in Indica rice cultivar by the slag amendment (Fig. 2). The interaction of the slag and cultivar did not significantly affect the relative abundance of dominant genera (Table S2).

Much dissimilarity was observed among the treatments at the species level (Supplementary material). The LD slag amendment triggered the proliferation of some dominant species which are mostly copiotrophic, and/or have a role in plant growth promoting activities (e.g., N_2_ fixation, phytohormone production), iron reduction, and versatility with respect to carbohydrate utilization (Table S3). Whereas among the dominant species, species like *Nitrospira moscoviensis*, *Candidatus Scalindua brodae*, and *Thauera linaloolentis* which have a potential role in nitrite-oxidation, anaerobic ammonium oxidation (anammox), and denitrification, respectively were markedly decreased due to the LD slag amendment (Table S3).

The LD slag application significantly (*P* < 0.05) increased the Margalef’s richness and Shannon Diversity indices, but significantly (*P* < 0.05) decreased the Pielou’s evenness in both rice cultivars. However, no significant variation of the measured diversity indices within the cultivars was found (Table 3).

**Table 3:** The LD slag amendment impacts on bacterial α-diversity indices.

	Japonica		Indica	
	Control	+ LD slag	Control	+ LD slag
Margalef’s richness	736 b	771 a	744 b	788 a
Pielou’s evenness	0.87 a	0.82 b	0.85 a	0.80 b
Shannon Diversity	8.19 b	8.46 a	8.25 b	8.52 a

Means of three replicates per treatment are presented. In a row under different rice cultivars means followed by a common letter are not significantly different at *P* < 0.05.

### The predictor variables of rhizosphere bacterial community

**Figure 3:**
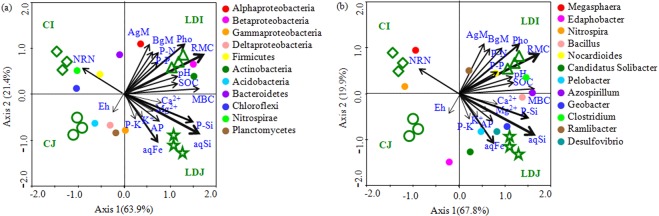
Canonical correspondence analysis (CCA) relating selected soil and plant variables to major phylogenetic groups (**a**) and genera (**b**). The resulting ordination biplot approximated the weighted average of each group/taxa with regard to each of the measured variables, which are represented as arrows. The lengths of these arrows indicate the relative importance of measured variables, whereas the angle between the arrows and the axis reflects the degree to which they are correlated. To statistically evaluate the significance (*P* < 0.01) of the first canonical axis and of all canonical axes together, the Monte Carlo permutation full model test with 999 unrestricted permutations was performed. MBC, Microbial biomass C; SOC, soil organic C; RMC, readily mineralizable C; NRN, ninhydrin nitrogen content; AP, available P, _aq_, water soluble; Pho, photosynthesis; AgM, Aboveground biomass; BgM, Belowground biomass; and P-N, P-P, P-K, and P-Si are straw N, P, K, and Si uptake, respectively.

**Figure 4:**
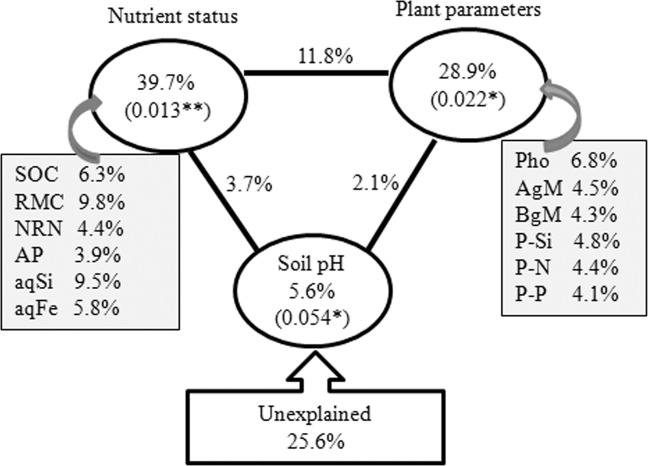
Variation partitioning analysis (VPA) of rhizosphere bacterial communities among important plant parameters, soil nutrient status, soil pH, and their interactions. The values in parentheses are *P*-values. Abbreviations for the measured variables are provided in Fig. 3.

Correlation analyses demonstrated that the soil pH, SOC, RMC, MBC, NRN, AP, aqSi, aqFe, photosynthetic rate, above and belowground biomass, and N, P and Si uptake (straw only) were significantly correlated with rhizosphere bacterial communities (Table 4). The CCA analysis depicted that the rhizosphere bacterial communities of slag amendment and without amendment treatments were distantly grouped (Fig. 3). The phyla Alphaproteobacteria, Betaproteobacteria, Actinobacteria, and the genera *Bacillus*, *Pelobacter*, *Azospirillum*, *Geobacter*, *Clostridium*, *Ramlibacter*, and *Desulfovibrio* were significantly and positively correlated with SOC, RMC, MBC, aqSi, photosynthetic rate, aqFe, AP, straw Si, N, and P uptake, and above and belowground biomass, whereas the phyla Nitrospirae, Chloroflexi and the genera *Megasphaera* and *Nitrospira* were significantly and positively correlated with NRN (Fig. 3, Table S4). The VPA analysis revealed that a set of soil nutrient attributes (i.e., SOC, RMC, NRN, AP, aqSi, and aqFe), plant attributes (i.e., photosynthesis, straw Si, N, and P uptake, and above- and belowground biomass), and soil pH accounted for 39.7, 28.9, and 5.6% of rhizosphere bacterial community variations, while 25.6% of the variation was unexplained (Fig. 4). Notably, nutrient status, plant attributes and soil pH had significant contribution to the variation in the rhizosphere bacterial community (Fig. 4).

**Table 4:** Correlation coefficient (*r*) between soil variables and bacterial community determined by Mantel test^†^.

Soil variables	Overall	Slag	Control
	*r* value	*r* value	*r* value
Soil pH	0.38*	0.56**	0.16 ns
Soil Eh	0.06 ns	0.03 ns	0.11 ns
SOC	0.44*	0.46*	0.24 ns
MBC	0.49*	0.54**	0.34*
RMC	0.62**	0.65**	0.36*
NRN	−0.38 *	−0.39*	0.11 ns
AP	0.34*	0.36*	0.08 ns
_aq_Si	0.59**	0.67**	0.38*
_aq_Fe	0.43*	0.51**	0.35*
K^+^	0.11 ns	0.18 ns	0.06 ns
Ca^2+^	0.24 ns	0.26 ns	0.13 ns
Mg^2+^	0.13 ns	0.15 ns	0.11 ns
Photosynthetic rate	0.54**	0.42 *	0.23 ns
Above ground biomass	0.37*	0.36*	0.22 ns
Belowground (Root) biomass	0.34*	0.39*	0.43*
Straw N uptake	0.43*	0.44*	0.39*
Straw P uptake	0.33*	0.34*	0.31*
Straw K uptake	0.19 ns	0.11 ns	0.24 ns
Straw Si uptake	0.56**	0.68**	0.35*

^†^Permutations, 9,999 (using the relative abundances of OTU as input in the analysis).

**Significant at *P* < 0.01, *Significant at *P* < 0.05, ns not significant. Abbreviations for soil variables are given in Table 1.

## Discussion

Since, the LD slag is rich in fertilizer components, its application remarkably increased nutrients (mostly C, P, Si, Fe, and Mg) in soil, consequently nutrients (N, P, and Si) acquisition by the plant and the crop yield. A favorable soil pH (towards neutral) under the slag amendment might enhance nutrient mineralization^11^. Silicon-induced higher plant photosynthesis and probably the resistance of rice plants to biotic and abiotic stress^1,5^ could be another reason for higher crop yield under the slag amendment. Increased shoot biomass likely increased plant N acquisition, which in turn decreased soil N availability in slag amendment treatments.

The increased activities of most of the soil enzymes having a role in carbon, nitrogen, and phosporous cycling indicated increased soil nutrient turnover under the slag amendment. The increased activities of soil enzymes could be explained by increased substrate availability and microbial biomass in response to the slag amendment^25^. The more labile carbon availability through Si-induced root exudation^5,13^ might have induced an increase in labile carbon degrading enzymes compared to recalcitrant carbon degrading enzymes.

This study evidenced that the rhizosphere bacterial communities were more rich and less even under the slag amendment, irrespective of rice cultivars (Table 2). The nutrient availability under the slag amendment might enhance r-strategic bacteria, resulting in more uneven bacterial communities that flourish more adequately under the increased availability of accessible nutrients^26^. However, this result should not be extrapolated to generalize that bacterial community under the slag amendment is consistently richer and less even over the whole cropping season, because in this study, bacterial community and diversity were estimated after harvesting. Moreover, since, rare species can also strongly influence ecosystem functioning, it is difficult to interpret the shift in richness and evenness with respect to crop management practices^27^. Hence, we focus our discussion on the shift in bacterial communities and the significantly influenced bacterial taxa which play a beneficial or detrimental role in the agro-ecosystem.

Proteobacteria were the most dominant phyla which significantly increased in slag amendment treatments compared with unamended treatments, irrespective of rice cultivars. The increase in relative abundance of Proteobacteria is likely due to the copiotrophic nature of the phyla which has been shown to proliferate under greater carbon availability^26^. Within Proteobacteria, Betaproteobacteria and Alphaproteobacteria were significantly increased in the slag amendment treatments. It has been postulated that Betaproteobacteria respond promptly to carbon availability, and are among the dominant inhabitants of paddy rhizosphere^28,29^. The members of the class Alphaproteobacteria are commonly known for their sole role in N_2_-fixation^30^ and their increase indicates that the LD slag amendment can be considered to stimulate N_2_-fixing ability of paddy soil. The higher photosynthesis and shoot biomass could enhance plant N uptake and thus decline available N in soil, in slag amendment treatments. The N deficiency is supposed to stimulate N_2_-fixing microorganisms in soil amended with the slag. Next to Proteobacteria, the phylum Actinobacteria significantly increased under the slag amendment. Actinobacteria play an important role in organic matter decomposition and biological buffering of soils^31,32^. Their relative increase probably facilitates nutrient mobilization in slag amended soils. However, discrepancy exists with regards to the occurrence of Actinobacteria in C-limited and -enriched ecosystems^17,19^. Unlike Proteobacteria and Actinobacteria, Acidobacteria, Bacteroidetes, Nitrospirae, and Chloroflexi were significantly decreased due to the slag fertilizer application. The decrease in relative abundance of Acidobacteria is likely due to the oligotrophic nature of the phylum and a strong negative correlation with soil pH^26^. Becteroidetes are in general considered to have an important role in the degradation of complex organic matter, especially polysachharides^33^. The higher labile carbon availability through increased root exudation under the slag amendment could diminish the role of Bacteroidetes to degrade complex organic matter. The low relative abundance of Nitrospirae, and Chloroflexi may be due to their adaptation to nitrogen limitation and flexibility for the limiting nutrient under slag amendment conditions^34^. In contrast to our findings, Bacteroidetes and Chloroflexi have been found to dominate in C-rich and N-rich environments, respectively^15,18^. Consequently, whereas there is a consensus that agronomic management practices alters soil microbial communities, the response of individual groups appears to be very context-specific and cannot be generalized across various agro-ecosystems^27^. The response is mostly dependent on soil physicochemical changes induced by the agronomic practices, which again differ among different soil types and under different climatic conditions.

Structural redundancy is widespread at higher taxonomic level. Taxonomy compositions at the lower (genus and species) level can differ significantly^35^. In this study, genera *Geobacter* and *Pelobacter* and species *Geobacter pickeringii*, *Thermovenabulum ferriorganovorum*, and *Magnetospirillum magnetotacticum* which are well known as Fe-reducer remarkably increased in response to the slag amendment (Table S3). Their increase might be due to greater Fe availability in slag amendment treatments. The increase in Fe-reducing bacteria under the slag amendment indicates that these bacteria could have a role in alleviating Fe-toxicity in the slag amended paddies. Species such as *Clostridium caenicola*, *Clostridium termitidis*, and *Caldilinea tarbellica* which are reported to have a role in carbohydrate utilization noticeably increased under the slag amendment, indicating that the slag amendment could stimulate C-degrading bacteria (Table S3). Likely, members of some dominant genera such as *Bacillus*, *Azospirillum*, and *Clostridium* and species such as *Bacillus arbutinivorans*, *Bacillus aryabhattai*, *Bacillus pumilus*, *Bacillus niacin*, and *Azospirillum zeae*, which have an important role in plant growth promoting (PGP) activities (Phytohormone production, P solubilization and N_2_-fixation) significantly increased under slag amendment treatments, indicating that the LD slag amendment in rice paddies could be promising to enhance PGP activities and hence crop yield.

A greater understanding of the regulatory factors (drivers) shaping rhizosphere microbiome is essential to use microbial technology for sustainable agriculture^19^. In agreement with our first hypothesis of this study, the slag amendment significantly increased nutrient availability in soil and consequently nutrient acquisition by the plant. These changes altered rhizosphere bacterial community composition, stimulated certain microbial taxa and induced soil enzyme activities. The soil nutrient availability and plant nutrient acquisition accounted for 39.7% and 13.3% of rhizosphere bacterial community variations (Fig. 4). In addition to soil nutrient fluxes, plant nutrient acquisition has been reported to alter soil microbial community composition^16,19^. According to our second hypothesis, the slag amendment remarkably increased Si concentrations in soil pore-water and likely enhanced photosynthesis and plant biomass. A significant contribution (15.6%) of plant attributes (photosynthesis and above and below ground biomass) to the variation in the rhizosphere bacterial community was observed. The rhizodeposition of organic C by the plant likely stimulated copiotrophic bacterial communities. Moreover, activities of certain oligotrophs could be stimulated by rhizodepoited-carbon, that further causes short term changes in SOM turnover by mineralizing the recalcitrant SOM, using fresh organic C as a source of energy^14^. In line with our third hypothesis, a significant contribution of soil pH to the variation in the rhizosphere bacterial community was observed. An increase in soil pH towards neutral value due to the slag amendment likely improved nutrient mobilization and thus microbial activities. Soil pH was identified as an important factor structuring microbial communities of agroecosystems^36^. Noteworthy, large differences in nutrient uptake between Indica and Japonica rice species, even without the slag amendment were observed. Rice species or cultivar variation has been reported to alter rhizosphere microbial community composition and nutrient cycling^37,38^. Plant species with high growth rate have higher rates of root exudation and labile compounds that can fuel rhizosphere microbial community^37,38^. The mantel test analysis revealed that RMC, MBC, aqSi, aqFe, straw and root biomass, and N, P, and Si uptake (straw only) significantly correlated with rhizosphere bacterial communities in control (without slag) treatments (Table 4).

In conclusion, the short-term LD slag amendment in rice cropping systems enhanced microbial biomass and activities, which increased nutrient availability in soil and nutrient uptake by the plant, finally improving crop yield. Our recent study also witnessed no heavy metal contamination in rice grain in response to short-term LD slag amendment^11^. The application of slag in the long term may increase soil alkalinity and accumulate heavy metals in soils, which may hinder soil microbial communities and functions and thus the crop yield. Long-term studies in different soil and environmental conditions are essential to clearly know the changes in soil microbial communities, their functional roles, and the crop yield under slag-based soil amendments in agriculture.

## Materials and Methods

### Characterization of steel slag

The Linz-Donawitz converter (LD) slag, generated from the basic oxygen furnace (BOF) process in steel manufacturing was obtained from POSCO, Pohang, South Korea. The LD slag was dried and ground to powder form. Concentrations of heavy metals in the slag were analyzed by following the methods described by the U.S EPA Hazardous Waste (USEPA SW-846). The USEPA Method 3050B was used to detect environmentally available metal constituents in the slag. The mobility of metals in the slag under neutral and acidic conditions was assessed by American Standards for Testing Materials (ASTM) Method D 3987 and the Toxicity Characteristic Leaching Procedure (TCLP) test using the USEPA Method 1311, respectively.

### Experimental design

The pot experiment was performed in a greenhouse at the agricultural farm of Gyeongsang National University, Jinju, Republic of Korea. The soil was collected from the surface layer (20 cm depth) of the experimental rice field at Gyeongsang National University (36°51’N, 128°28’E). The selected soil belonged to the Jisan series (fine-silty, mixed mesic family of Fluventic Typic Haplaquepts) and had the following initial chemical characteristics before the experiment; pH 5.6 (1:5 with H_2_O), total soil organic C (SOC) 25.8 g kg^−1^, total N (TN) 2.76 g kg^−1^, available P_2_O_5_ (AP) 76 mg kg^−1^, and available SiO_2_ 86 mg kg^−1^. The pH and concentrations of SiO_2_ of the current field soil is much lower as compared with that of Korean recommendation for rice paddies. The recommended value for soil pH and SiO_2_ content is 6.0-6.5 and 130-180 mg kg^−1^, respectively^39^.

Collected soil samples were air dried, sieved (5 mm) and packed into the Wagner pots (24 cm in diameter and 30 cm in height) with a bulk density of 1.2 g cm^−3^. The LD slag was added at the rate of 2 Mg ha^−1^ into each individual pot, 3 days prior to rice transplanting and pots were flooded immediately. The basal NPK synthetic fertilizers comprising urea (55 kg N ha^−1^), fused superphosphate (45 kg P_2_O_5_ ha^−1^), and potassium chloride (40 kg K_2_O ha^−1^) were applied one day prior to the rice transplanting, whereas 22 kg N ha^−1^ was applied at the tillering stage (2 weeks after rice transplanting), and 33 kg N ha^−1^ and 18 kg K_2_O ha^−1^ was applied at the panicle initiation stage (6 weeks after rice transplanting). Slag and fertilizers were applied as per standard Korean recommendations for rice cultivation^39^. Twenty-five (25) days old seedling of rice with two different cultivars (Rc158; Indica and Dongjinbyeo; Japonica) but the same growth duration (120 days) were transplanted at two seedlings per pot. The pots were similarly maintained under submerged condition (5 cm above the soil surface) throughout the experiment. The experiment was conducted by installing three replicates for each treatment under greenhouse conditions.

### Sampling and analysis

Soil samples at harvesting were collected from each replicated pots. Soil redox potential (Eh) and pH were measured by Eh meter (PRN-41, DKK-TOA Corporation) and pH meter (Orion 3 star, Thermo Electron Corporation), respectively. Soil organic C (SOC) was determined by dry combustion method (TOCA, Shimadzu) and total nitrogen (TN) was measured using the micro-Kjeldahl method^40^. Readily mineralizable carbon (RMC), and nitrogen (NRN) content was measured by the method of Inubushi *et al*.^41^. The available P was estimated by the molybdenum blue method^42^. Microbial biomass carbon (MBC) was measured by the modified chloroform fumigation extraction method^43^. Soil exchangeable K^+^, Ca^2+^, and Mg^2+^ were extracted with 1 N NH_4_CH_3_CO_2_ (pH 7.0) and analyzed by atomic absorption spectrophotometer (Shimadzu 660, Kyoto).

The soil pore-water was collected in the vials by using the rhizo-sampler (EcoTech^®^, Germany). The vials were washed carefully with acids, flushed with N_2_ and finally were crimp sealed before the sampling. The pore-water Si (aqSi) and Fe (aqFe) concentrations were measured by inductively coupled plasma-optical emission spectrometry (ICP-OES), (Vista-MPX, Varian, Australia)^23^.

A portable photosynthesis system (CIRAS-2, PP-Systems, UK) was used to measure the photosynthetic rate of leaf from 9:00 am to 10:00 am. Different growth stages of rice, including maximum tillering, heading and grain filling were selected to measure the photosynthetic rate.

At rice harvest, rice straw was collected and oven-dried at 70 °C until constant weight. To measure N, P, K, and Si concentration in plant tissue, the straw was weighted and ground to powder. Nitrogen, P, and K concentrations in the straw were measured by standard methods as described by Coung *et al*.^44^. The Si concentrations in rice straw were measured by the dilute hydrofluoric acid extraction and spectrometric molybdenum method^45^.

For microbial analysis, the rhizosphere soil (soil that tightly adhered to the root) was collected by rinsing the roots in sterile double distilled water (ddH_2_O) and then centrifuged at a high speed (10,000 ×g for 10 min).

### Enzyme fingerprinting using APIZYM assay

The relative activities for soil enzyme were measured by a semi-quantitative APIZYM system (Biomerieus, USA) according to the manufacturer’s protocol^16,46,47^. The APIZYM kit supplied with 20 microcupules, contains dehydrated chromogenic substrates for different hydrolytic enzymes and control (Supplementary Table S5). Fresh rhizosphere soils were mixed with sterile nonopure water (1: 1.25 w-v), homogenized in a horizontal shaker for 10 min, centrifuged at 2000g for 15 min and the supernatant was collected for analysis of soil enzymatic activities. Supernatants (70 μl) were dispensed into each microcupules and incubated at 32°C for 24 h. After that, the reagents ZYM A and ZYM B (30 μl of each) were added to each microcupule and left for 5 minutes for color development. The color development was evaluated and a numerical value ranging from 0 to 5 was assigned according to the color chart provided by the manufacturer.

### DNA extraction, barcoded PCR, and illumina sequencing

The total genomic DNA was extracted from fresh rhizosphere soil (0.5 g) using a FastDNA SPIN kit for soil (MP Biomedicals, USA), following the manufacturer’s instructions. PCR amplification targeting the V4-V5 region of the 16 S rRNA genes was carried out using a primer set 515 F/926R^48^, with a 12 bp bar code on the reserve primer. Each PCR mixture (25 µL) contained 0.2 U AccuPrime High Fidelity Taq Polymerase, 2 µL 10× AccuPrime PCR buffer II (including dNTPs) (Invitrogen, USA), 0.4 mM of both forward and reverse primers, and 5 ng of DNA. PCR reaction was performed under the condition of an initial denaturation at 95°C for 3 min followed by 28 cycles of 95°C for 45 s, 50°C for 45 s, 68°C for 90 s, and a final extension of 68°C for 5 min. For each sample, triplicate PCR amplicons were pooled and gel purified using Qiaquick gel extraction kit (Qiagen). The amplicons were mixed in equimolar concentrations and the library was submitted for a single run of paired-end sequencing on the Illumina MiSeq platform with the MiSeq Reagent v2 kit at Macrogen, South Korea.

### Sequence processing and taxonomic assignment

Raw sequences were processed and analyzed using QIIME pipeline^49^. Sequences were quality filtered for mismatches with barcodes, homopolymers, ambiguous bases, and chimera as described previously^15^. Operational taxonomic units (OTUs) were identified at a threshold level of 97% sequence similarity using UCLUST^50^. The phylogenetic classification of the reads was performed using Ribosomal Database Project (RDP) classifier with the Greengenes database v13.8 at 80% confidence threshold^51^. The species-level classification was generated by ‘ClassifyReads’ an algorithm available in MiSeq Reporter Metagenomics Workflow^15^. Alpha diversity index, i.e., Shannon diversity index, Margalef’s Richness and Pielou’s Evenness were estimated using Mothur (v.1.28.0).

### Statistical analysis

The values of all analyses are presented as arithmetic means ± standard deviation (SD) with n = 3, unless otherwise indicated. Statistically significant differences were tested using ANOVA followed by Tukey’s honestly significant difference (HSD) test. Before statistical analysis, all dependent variables were tested for normality using the Kolmogorov-Smirnov test and log-transformed if necessary. The difference in bacterial community composition (Bray-Curtis dissimilarities) was assessed by permutational multivariate analysis of variances (PERMANOVA). To demonstrate the changes in relative abundance of bacteria, the response ratio (RR) was estimated and to determine whether the response ratio was significantly different from zero, one-sample t-test was conducted. The relationship between the rhizosphere bacterial community and soil/plant variables were assessed by conducting mantel test, variation partitioning analysis (VPA), and canonical correspondence analysis (CCA). The analyses were performed using functions in the Vegan package (v. 1.15-1) in R v. 2.8.1^52^.

## Supplementary information

Supplementary information.

## Data Availability

The 16S-rRNA gene sequences obtained from this study are available in the GenBank Sequence Read Archive with accession number SRP136428 and under BioProject PRJNA445617.
