# Supplementary material for: Steel slag amendment impacts on soil microbial communities and activities of rice (Oryza sativa L.)
Source: Sci Rep. 2020 Apr 21;10:6746. doi: 10.1038/s41598-020-63783-1 (PMC7174330; doi:10.1038/s41598-020-63783-1)
Supplement: Supplementary file 1 — Supplementary information. [file 41598_2020_63783_MOESM1_ESM.pdf]

Supporting information

Steel slag amendment impacts on soil microbial communities and activities of rice (*Oryza sativa* L.)

Suvendu Das [1], Hyo Suk Gwon [2], Muhammad Israr Khan [2], Seung Tak Jeong [2], Pil Joo Kim [1, 2,*]

[1] Institute of Agriculture and Life Sciences, Gyeongsang National University, Jinju, 660-701, Republic of Korea

[2] Division of Applied Life Science, Gyeongsang National University, Jinju, 660-701, Republic of Korea

[*]Corresponding Author: Pil Joo Kim, E-mail: pjkim@gnu.ac.kr

Phone: +82-55-751-5466; Fax: +82-55-757-0178 14

Table S1. Chemical characteristics of LD slag

Metal	Concentration (mg kg^{-1})	ASTM (mg L^{-1})	TCLP (mg L^{-1})	TCLP screening criterion (mg L^{-1}) [a]	Exceed criterion ?
As	0.603	ND	0.029	5.0	No
Ba	124	0.4	4.17	100.0	No
Cd	1.38	ND	0.023	1.0	No
Cr	23.8	ND	0.652	5.0	No
Pb	6.03	0.004	0.084	5.0	No
Hg	ND	ND	ND	0.2	No
Se	9.51	ND	0.244	1.0	No
Ag	3.81	0.006	0.252	5.0	No
Sb	0.59	ND	ND		
Mn	38126	0.723	160		
Ni	17.1	ND	0.425		
Zn	59.0	0.004	2.26		
Co	0.92	ND	ND		
Al	31969				
Ca	309950				
Fe	128751				
Mg	1029				
Si	92684				

[a] From Method 1311 TCLP
Values are normal 95% UCL (upper confidence limit of the mean) concentrations.
ASTM, American Standards for Testing Materials, TCLP, Toxicity Characteristic Leaching Procedure, ND, Not detected.

Table S2. PERMANOVA statistics for slag, cultivar, and slag × cultivar factors for major (a) bacterial groups and (b) genus shown in Figure 1. Letters in bold are significant.

	Slag		Cultivar		Slag × Cultivar	
	F-ratio	*P* value	*F*-ratio	*P* value	*F*-ratio	*P* value
(a)						
Proteobacteria	**23.6**	**0.00001**	0.89	0.43	0.13	0.83
Alphaproteobacteria	**21.2**	**0.00001**	1.11	0.39	0.11	0.85
Betaproteobacteria	**24.1**	**0.00001**	0.83	0.46	0.15	0.81
Gammaproteobacteria	1.23	0.32	0.52	0.51	0.06	0.94
Deltaproteobacteria	1.19	0.37	0.55	0.54	0.04	0.97
Firmicutes	3.26	0.18	0.47	0.56	0.11	0.85
Actinobacteria	**34.6**	**0.00001**	0.85	0.45	0.22	0.74
Acidobacteria	**27.4**	**0.00001**	0.88	0.43	0.19	0.78
Bacteroidetes	**22.8**	**0.00001**	0.81	0.47	0.13	0.83
Chloroflexi	**54.5**	**0.00001**	1.56	0.26	0.36	0.68
Nitrospirae	**43.7**	**0.00001**	1.24	0.33	0.27	0.65
Planctomycetes	1.26	0.29	0.92	0.41	0.05	0.96
(b)						
Megasphaera	**43.6**	**0.00001**	1.92	0.17	0.51	0.48
Edaphobacter	**36.5**	**0.00001**	1.66	0.21	0.46	0.57
Nitrospira	**38.4**	**0.00001**	1.76	0.19	0.47	0.56
Bacillus	**46.5**	**0.00001**	1.93	0.16	0.53	0.47
Nocardioides	**6.8**	**0.017**	**3.06**	**0.05**	0.13	0.84
Candidatus Solibacter	**31.2**	**0.00001**	1.26	0.91	0.44	0.59
Pelobacter	**7.8**	**0.008**	**3.16**	**0.04**	0.14	0.82
Azospirillum	**39.4**	**0.00001**	1.78	0.21	0.48	0.55
Geobacter	**43.2**	**0.00001**	1.91	0.17	0.51	0.48
Clostridium	**37.6**	**0.00001**	1.75	0.19	0.47	0.57
Ramlibacter	**9.3**	**0.005**	**3.13**	**0.04**	0.11	0.84
Desulfovibrio	**6.6**	**0.019**	2.88	0.07	0.08	0.91

Table S3. Effects of the LD slag amendment on some of the dominant identified species and their potential role. (all identified species are provided in supplementary exel file)

Species	CJ		LDJ		CI		LDI		Role	Reference
	No. of sequences	Relative abundance (%)	No. of sequences	Relative abundance (%)	No. of sequences	Relative abundance (%)	No. of sequences	Relative abundance (%)		
Magnetospirillum magnetotacticum	938	0.25	13627	3.42	663	0.19	3736	0.92	Fe(III) reduction	Nogucgi et al., 1999
Thermovenabulum ferriorganovorum	368	0.10	851	0.21	775	0.23	1642	0.40	Fe(III) reduction	Ogg et al., 2010
Geobacter pickeringii	358	0.098	1694	0.42	1062	0.30	3011	0.72	Fe(III) reduction	Shelobolina et al., 2007
Caldilinea tarbellica	583	0.16	1108	0.28	792	0.23	2378	0.57	Vartatile C degradation	Gregoire et al., 2011
Clostridium caenicola	660	0.18	2291	0.57	276	0.08	941	0.23	Cellulose degradation	Shiratori et al., 2009
Clostridium termitidis	371	0.10	1290	0.32	279	0.08	854	0.21	Cellulose degradation	Hethener et al., 1992
Bacillus aryabhattai †	907	0.25	2289	0.74	1352	0.40	3008	0.73	PGPR	Park et al., 2017
Bacillus arbutinivorans †	302	0.08	721	0.18	515	0.15	902	0.22	PGPR	Yadav et al., 2011
Bacillus pumilus †	191	0.05	821	0.20	282	0.08	771	0.19	PGPR	Maneroa et al., 2001
Bacillus niacin ‡	339	0.09	817	0.19	473	0.14	1082	0.26	PGPR	Kim et al., 2011
Azospirillum zeae	231	0.06	1198	0.28	322	0.09	896	0.22	N_2-fixation	Mehnaz et al., 2007
Azospirillum rugosum	88	0.02	902	0.22	37	0.01	817	0.19	N_2-fixation	Lai et al., 2008
Nitrospira moscoviensis	1236	0.33	332	0.05	1343	0.40	352	0.08	Nitrite oxidation	Ehrich et al., 1995
Candidatus Scalindua brodae	1243	0.34	355	0.09	2448	0.71	404	0.09	Annamox	Schmid et al., 2003
Thauera linaloolentis	4805	1.31	26	0.007	1168	0.33	74	0.02	Denitrification	Foss et al., 1998

† Plant growth promoting rhizobacteria (PGPR), Phytohormones production.
‡ PGPR, P solubilization,

Table S4. Spearman correlation coefficients between the relative abundance of major phylogenetic groups and soil variables (a), and major genera and soil variables (b) as determined by Mantel tests

(a)

	MBC	SOC	RMC	NRN	AP	aqSi	aqFe	K^+	Ca^{2+}	Mg^{2+}	pH	Eh	Pho	AgM	BgM	P-N	P-P	P-K	P-Si
Proteobacteria	0.72**	0.63*	0.78**	-0.67**	0.61*	0.69**	0.48*	0.19	0.31	0.13	0.31	-0.04	0.71**	0.63*	0.52*	0.66*	0.58*	0.11	0.70**
Alphaproteobacteria	0.70**	0.59*	0.74**	-0.57*	0.52*	0.60*	0.39*	0.08	0.26	0.02	0.47*	-0.12	0.69**	0.70**	0.61*	0.59*	0.41*	0.06	0.66*
Betaproteobacteria	0.73**	0.64*	0.79**	-0.67**	0.60*	0.67**	0.46*	0.18	0.41*	0.12	0.44*	-0.12	0.73**	0.65*	0.54*	0.63*	0.45*	0.16	0.61*
Gammaproteobacteria	0.24	0.13	0.03	-0.06	0.13	0.14	0.38*	0.06	0.11	0.06	-0.10	0.23	-0.12	-0.23	-0.31	0.19	0.15	0.13	0.13
Deltaproteobacteria	-0.16	-0.07	-0.06	-0.01	0.08	0.06	0.22	0.02	0.05	0.02	-0.23	0.24	-0.23	-0.58*	-0.68**	-0.15	0.07	0.05	0.05
Firmicutes	0.45*	0.49*	0.46*	0.31	-0.55*	-0.59*	-0.55*	-0.17	-0.16	-0.14	-0.16	-0.17	-0.42*	-0.13	-0.16	0.46*	-0.19	-0.18	-0.41*
Actinobacteria	0.69**	0.65*	0.76**	-0.64*	0.63*	0.65*	0.42*	0.14	0.22	0.17	0.26	-0.22	0.79**	0.68**	0.58*	0.67*	0.39*	0.21	0.56*
Acidobacteria	-0.28	-0.38*	-0.43*	0.16	-0.07	-0.18	-0.01	-0.01	-0.16	-0.03	-0.53*	0.28	-0.61*	-0.74**	-0.73**	-0.24	-0.06	-0.05	-0.14
Bacteroidetes	-0.11	-0.13	-0.01	0.21	-0.13	-0.19	-0.50*	-0.11	-0.17	-0.11	0.21	-0.14	0.04	0.25	0.41*	-0.19	-0.13	-0.11	-0.17
Chloroflexi	-0.68**	-0.48*	-0.72**	0.71**	-0.72**	-0.71**	-0.61*	-0.18	-0.27	-0.19	-0.23	0.13	-0.51*	-0.48*	-0.28	-0.61*	-0.16	-0.20	-0.23
Nitrospirae	-0.71**	-0.51*	-0.77**	0.76**	-0.74**	-0.73**	-0.64*	-0.19	-0.43*	-0.37*	-0.19	0.08	-0.53*	-0.47*	0.23	-0.55*	-0.18	-0.16	-0.13
Planctomycetes	-0.13	-0.18	-0.21	0.01	-0.01	-0.07	0.05	0.03	-0.09	-0.01	-0.12	0.21	-0.41*	-0.68**	-0.62*	-0.13	-0.06	0.07	-0.05

(b)

	MBC	SOC	RMC	NRN	AP	aqSi	aqFe	K^+	Ca^{2+}	Mg^{2+}	pH	Eh	Pho	AgM	BgM	P-N	P-P	P-K	P-Si
Megasphaera	-0.66*	-0.58*	-0.61*	0.74**	-0.63*	-0.76**	-0.73**	-0.18	-0.54*	-0.19	-0.11	-0.03	-0.54*	-0.09	-0.02	-0.57*	-0.45*	-0.17	-0.68**
Edaphobacter	-0.11	-0.23	-0.26	0.09	-0.09	-0.13	0.07	-0.07	-0.13	-0.02	-0.28	0.22	-0.50*	-0.51*	-0.45*	-0.16	-0.05	-0.08	-0.17
Nitrospira	-0.76**	-0.53*	-0.76**	0.78**	-0.67**	-0.64*	-0.64*	-0.12	-0.57*	-0.19	-0.30	0.08	-0.70**	-0.31	-0.28	-0.48*	-0.48**	-0.14	-0.59*
Bacillus	0.65**	0.65*	0.77**	-0.71**	0.68**	0.73**	0.65*	0.19	0.23	0.16	0.37*	-0.04	0.72**	0.42*	0.31	0.56*	0.51**	0.17	0.47*
Nocardioides	0.49*	0.44*	0.50*	-0.13	0.21	0.55*	0.14	0.06	0.09	0.08	0.22	-0.16	0.52*	0.44*	0.43*	0.49*	0.21	0.06	0.56*
C. Solibacter	0.28	0.23	0.10	-0.46*	0.27	0.44*	0.65*	0.17	0.18	0.17	0.13	0.15	0.08	-0.12	-0.28	0.16	0.24	0.17	0.44*
Pelobacter	0.29	0.26	0.27	-0.49*	0.31	0.56*	0.23	0.18	0.22	0.23	0.24	0.11	0.12	0.05	-0.11	0.17	0.14	0.14	0.56*
Azospirillum	0.71**	0.53*	0.73**	-0.78**	0.64*	0.67**	0.64*	0.19	0.31	0.26	0.26	-0.03	0.66*	0.36*	0.31	0.48*	0.47*	0.13	0.49*
Geobacter	0.73*	0.66*	0.75**	-0.69**	0.57*	0.71**	0.61*	0.13	0.29	0.24	0.23	-0.05	0.69**	0.58*	0.35*	0.42*	0.39*	0.19	0.56*
Clostridium	0.66**	0.71**	0.76**	-0.67**	0.53*	0.68**	0.56*	0.16	0.29	0.11	0.27	-0.04	0.71**	0.63*	0.41*	0.57*	0.44*	0.16	0.51*
Ramlibacter	0.61*	0.51*	0.64*	-0.41*	0.27	0.29	0.28	0.08	0.17	0.05	0.29	-0.19	0.29	0.23	0.26	0.47*	0.25	0.05	0.21
Desulfovibrio	0.53*	0.49*	0.65*	-0.70**	0.23	0.63*	0.57*	0.21	0.23	0.17	0.13	-0.01	0.58*	0.21	0.06	0.45*	0.22	0.18	0.56*

Significance: *, $p < 0.05$; **, $p < 0.01$. MBC, Microbial biomass C; SOC, soil organic C; RMC, readily mineralizable C; NRN, ninhydrin nitrogen content; AP, available P, $_{aq}$, water soluble; Pho, photosynthesis; AgM, Aboveground biomass; BgM, Belowground biomass; P-N, straw N uptaket; P-P, straw P uptake; P-K, straw K uptake; P-Si, straw Si uptake

Table S5. Overview of enzyme activities studied in soil using APIZYM assay.

	Process	Enzyme	EC number	Substrate
C cycle	Maltose degradation	α-glucosidase	3.2.1.31	2-naphthyl-2-D-glucopyranoside
	Cellobiose degradation	β-glucosidase	3.2.1.21	6-bromo-2-naphthol- α-D-galactopyranoside
	Melibiose degradation	α-galactosidase	3.9.1.1	6-Br-2-naphthyl- α-D-galactopyranoside
	Lactose degradation	β-galactosidase	3.2.1.22	2-naphthyl- α-D-galactopyranoside
	Mannose degradation	α-mannosidase	3.2.1.24	6-bromo-2-naphthyl-2-D-mannopyranoside
	Fructose degradation	α-fucosidase	3.2.1.51	2-naphthyl- αL-fucopyranoside
	Hemicelluloses degradation	β-glucuronidase	3.2.1.31	Naphthyl-AS-BI- βD-glucuronide
		Esterase	3.1.1.1	2-naphthyl-butyrate
		Lipase	3.1.1.3	2-naphthyl-myristate
	Polysaccharide degradation	N-acetyl-β-glucosaminidase	3.2.1.30	1-Naphthyl-N-acetyl-βD-glucosaminide
N cycle	N acquisition	Leucine-aminopeptidase	3.4.11.1	L-leucyl-2-naphthylamide
		Cystein-aminopeptidase	3.4.11.2	L-cystyl-2-naphthylamide
		Trypsin	3.4.11.3	N-benzol-DL-arginine-2-naphthylamide
		Chymotripsin	3.4.21.4	N-glutaryl-phenylalanine-2-naphthylamine
P cycle	P acquisition	Phosphohydrolase	3.1.3.2	Naphthyl AS-BI-phosphate
		Acid phosphomonoesterase	3.4.21.3	2 naphthyl-phosphate
		Alkaline phosphomonoesterase	3.1.3.1	2 naphthyl-phosphate

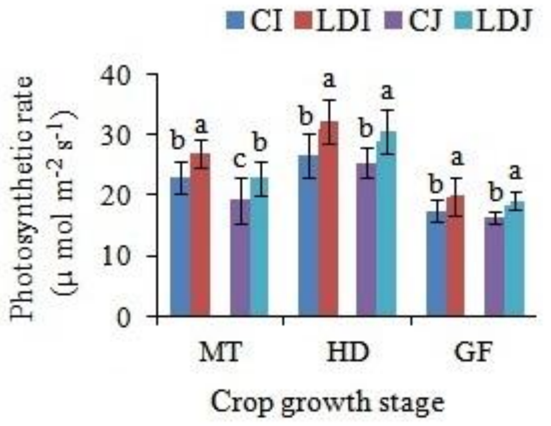

Fig. S1. Effects of LD slag amendment on photosynthetic rate of Japonica and Indica rice. In bar diagram, different letters within the treatments on different sampling days represent a significant difference ($p < 0.05$). MT, maximum tillering (42 DAT); HD, heading (70 DAT), GF, grain filling (105 DAT). CJ, Japonica rice cultivated with slag; LDJ, Japonica rice cultivated with slag; CI, Indica rice cultivated without slag; LDI, Indica rice cultivated with slag.

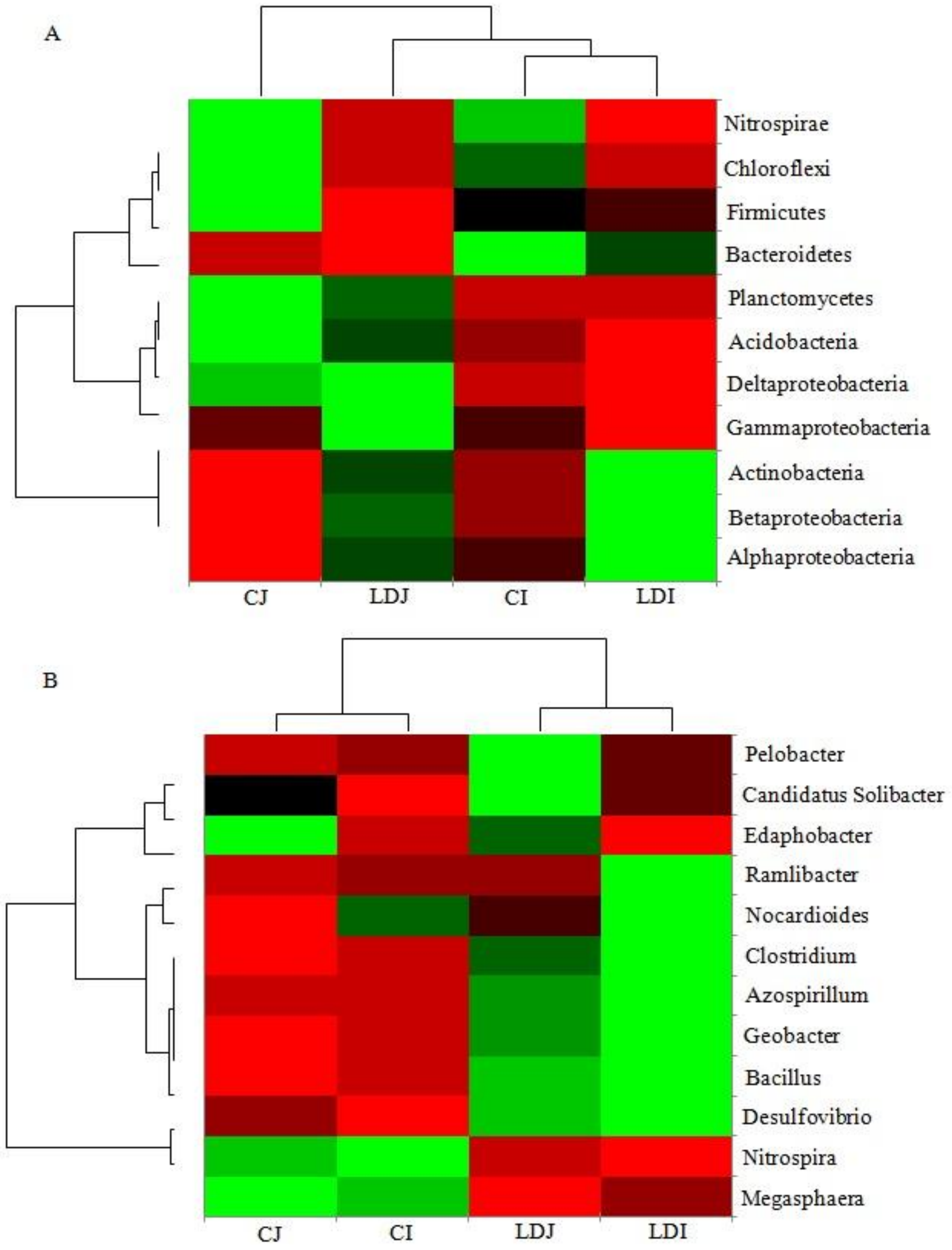

Fig. S2. Heatmap of A) major taxonomic groups, B) major genera. The relative abundance of each bacterial distribution is depicted by color intensity. The relative abundance bacteria in different samples is colored in shades of red (low relative abundance) to green (high relative abundance) through black.

References

Ehrich, S., Behrens, D., Lebedeva, E., Ludwig, W., & Bock, E. A new obligately chemolithoautotrophic, nitrite-oxidizing bacterium, *Nitrospira moscoviensis* sp. nov. and its phylogenetic relationship. *Arch. Microbiol.* **164(1)**, 16-23 (1995).

Foss, S., & Harder, J. *Thauera linaloolentis* sp. nov. and *Thauera terpenica* sp. nov., isolated on oxygen-containing monoterpenes (linalool, menthol, and eucalyptol) nitrate. *Syst. Appl. Microb* **21(3)**, 365-73 (1998)..

Gregoire, P. et al. *Caldilinea tarbellica* sp. nov., a filamentous, thermophilic, anaerobic bacterium isolated from a deep hot aquifer in the Aquitaine Basin. *Int. J. Syst. Evol. Microb.* **61**, 1436–1441 (2011).

Gutierrez-Maneroa, F.J., Ramos-Solanoa, B., Probanzaa, A., Mehouachib, J., Tadeob, F.R., & Talonb, M. The plant-growth-promoting rhizobacteria *Bacillus pumilus* and *Bacillus licheniformis* produce high amounts of physiologically active gibberellins. Physiol. *Planta* **111**, 206-211 (2001).

Hethener, P., Brauman, A., & Garcia, J.L. *Clostridium termitidis* sp. nov., a cellulolytic bacterium from the gut of the wood-feeding termite, *Nasutitermes lujae*. *Syst. Appl. Microb.* **15**, 52-58 (1992).

Kim, W. et al. Genetic diversity of cultivable plant growth-promoting rhizobacteria in Korea. *J. Microb. Biotec.* **21(8)**, 777-790 (2011).. https://doi.org/10.4014/jmb.1101.01031

Lai, W.A., Rekha, P.D., Arun, A.B., & Young, C.C. Effect of mineral fertilizer, pig manure, and *Azospirillum rugosum* on growth and nutrient contents of *Lactuca sativa* L. *Biol. Fert. Soils* **45**, 155-164 (2008).

Mehnaz, S., Weselowski, B., & Lazarovits, G. *Azospirillum zeae* sp. nov., a diazotrophic bacterium isolated from rhizosphere soil of Zea mays. *Int. J. Syst. Evol. Microb.* **57**, 2805–2809 (2007).

Nogucgi, Y., Fujiwara, T., Yoshimatsu, K., & Fukumori Y. Iron Reductase for Magnetite Synthesis in the Magnetotactic Bacterium *Magnetospirillum magnetotacticum*. *J. Bacteriol.* **181(7)**, 2142–2147 (1999).

Ogg, C.D., Greene, A.C., & Patel, B.K.C. *Thermovenabulum gondwanense* sp. nov., a thermophilic anaerobic Fe(III)-reducing bacterium isolated from microbial mats thriving in a Great Artesian Basin bore runoff channel. *Int. J. Syst. Evol. Microb.* **60**, 1079–1084 (2010).

Park, Y.G. et al. *Bacillus aryabhattai* SRB02 tolerates oxidative and nitrosative stress and promotes the growth of soybean by modulating the production of phytohormones**.** *PLoS One.* **12(3)**, e0173203 (2017).

Schmid, M. et al. *Candidatus* "*Scalindua brodae*", sp. nov., *Candidatus* "*Scalindua wagneri*", sp. nov., two new species of anaerobic ammonium oxidizing bacteria. *Syst. Appl. Microb.* **26(4)**, 529-38 (2003).

Shelobolina, E.S. et al. *Geobacter pickeringii* sp. nov., *Geobacter argillaceus* sp. nov. and *Pelosinus fermentans* gen. nov., sp. nov., isolated from subsurface kaolin lenses. *Int. J. Syst. Evol. Microb.* **57**, 126–135 (2007).

Shiratori, H. et al. *Clostridium clariflavum* sp. nov. and *Clostridium caenicola* sp. nov., moderately thermophilic, cellulose-/cellobiose-digesting bacteria isolated from methanogenic sludge. *Int. J. Syst. Evol. Microb.* **59**, 1764–1770 (2009).

Yadav, S., Kaushik, R., Saxena, A.K., & Arora, D.K. Diversity and phylogeny of plant growth-promoting bacilli from moderately acidic soil. *J. Basic Microb.* **51**, 98–106 (2011).
